# Text Messaging Between Patients With Inflammatory Rheumatic Diseases and Pharmacists to Solve Drug-Related Problems: Prospective Feasibility Study

**DOI:** 10.2196/66514

**Published:** 2025-10-08

**Authors:** Lex L Haegens, Charlotte L Bekker, Marcel Flendrie, Bart J F van den Bemt, Victor J B Huiskes

**Affiliations:** 1 Department of Research, Sint Maartenskliniek, Hengstdal 3, Ubbergen, 6574NA, The Netherlands, 31 683833157; 2 Department of Pharmacy, Radboudumc, Nijmegen, The Netherlands; 3 Department of Rheumatology, Sint Maartenskliniek, Ubbergen, The Netherlands; 4 Department of Pharmacy, Sint Maartenskliniek, Ubbergen, The Netherlands

**Keywords:** drug-related problems, text messaging, disease-modifying antirheumatic drug, inflammatory rheumatic diseases, digital health, feasibility, inflammatory, rheumatic diseases, DRPs, questionnaires, mHealth, mobile health

## Abstract

**Background:**

Patients with inflammatory rheumatic diseases often experience drug-related problems (DRPs). As these can result in negative health consequences, DRPs should be identified and addressed in a timely manner. Text messaging between patients and pharmacists at the initiative of the patient has the potential to deliver support with DRPs more continuously, increase accessibility and efficiency, and enhance patient involvement in the process of identifying and solving DRPs.

**Objective:**

This study aimed to assess the feasibility of text messaging from both the patients’ and health care practitioners’ perspectives before a large-scale implementation.

**Methods:**

Adult patients using a disease-modifying antirheumatic drug were given access to text messaging with pharmacists to discuss DRPs for a period of 8 weeks. Patients received a response from a pharmacist within 4 working hours. Feasibility was evaluated based on five domains of Bowen’s framework for designing feasibility studies: (1) demand: actual use, expressed interest (user version of the Mobile Application Rating Scale – section E), and factors impacting future use; (2) limited efficacy: number of DRPs solved, DRPs resulting in follow-up, and DRPs warranting involvement of health care provider; (3) implementation: degree of execution (number of conversations answered within service level) and resources needed (pharmacists’ time investment per conversation); (4) acceptability: satisfaction and appropriateness (theoretical framework of acceptability); and (5) practicality: ability to carry out intervention activities (System Usability Scale). Data were collected by means of usage data and a questionnaire.

**Results:**

In total, 45 patients (median age 57, IQR 52-65 y; n=31, 69% female) and 5 pharmacists (median age 41, IQR 26-47 y; n=1, 20% female) actively participated in this study. In the demand domain, 158 unique DRPs were raised in 133 conversations, with a median of 3 (IQR 2-4) unique DRPs per patient. Expressed interest was rated high by patients (median 4, IQR 4-5), and 90% (37/41) of patients would recommend text messaging to others. In the limited-efficacy domain, all DRPs were solved, and 77% (122/158) of DRPs warranted involvement of a health care provider. In the implementation domain, 87% (116/133) of conversations were answered within the promised timeframe with a median time investment of 4:15 (IQR 2:21-7:27) minutes per conversation. Acceptability was rated high by patients (median 4, IQR 4-5) and pharmacists (median 5, IQR 4-5). Finally, in the practicality domain, System Usability Scale was scored above average for patients (mean 72, SD 18) and pharmacists (mean 81, SD 16).

**Conclusion:**

Text messaging with pharmacists at the initiative of patients with rheumatic diseases seems feasible for discussing DRPs in terms of limited efficacy, implementation, acceptability, demand, and practicality for patients and pharmacists.

## Introduction

Pharmacotherapy is the cornerstone of the treatment of many inflammatory rheumatic diseases, which involves patients often using multiple drugs chronically. Disease-modifying antirheumatic drugs (DMARDs) are effective in decreasing disease activity and disability in patients with inflammatory rheumatic diseases and can even decrease or prevent joint damage [1]. For optimal treatment outcomes and patient safety, it is essential that DMARDs are used as agreed upon between a patient and health care provider, also called adherence [2]. However, nonadherence is common in this population [3], which is partly caused by drug-related problems (DRPs) experienced by patients with rheumatic diseases [4]. These DRPs encompass side effects, difficulties with medication management (eg, problems regarding opening medication packaging, administering and storing medication, or traveling with medication), a lack of knowledge, or concerns about lack of effect and unwanted effects of long-term medication use, among others. DRPs can potentially lead to negative health consequences, such as increased morbidity, which in turn can result in increased health care costs and decreased quality of life [5,6]. Thus, DRPs should be identified and resolved as soon as possible (ie, at the earliest stage of DRP development possible) to be able to prevent or minimize these negative consequences. However, during regular health care provider consultations, patients either do not always report all DRPs or health care providers do not act upon DRPs reported by patients [7]. In addition, patient-provider consultations occur only a few times a year, while DRPs can occur continuously, often in between these contact moments. Ideally, for adequate management of DRPs, patients should be able to contact their health care provider as soon as a DRP occurs, and health care providers should be able to react in a timely manner. To this end, one could argue that more continuous accessible contact between patients and health care providers is needed.

eHealth, which entails information and communication technology in health care that can benefit both patients and health care providers, could facilitate such contact by, for example, making health care more efficient and more easily accessible because of its time- and place-independent possibilities [8,9]. In addition, eHealth can increase patient involvement in the process of identifying and solving DRPs, for example, by lowering the threshold for patients to contact health care providers. Previous research has shown that patient involvement is important in identifying and solving clinically relevant DRPs [10,11], underlining the key role of patients in this process.

One such eHealth instrument is text messaging with a pharmacist regarding DRPs. Text messaging has the potential to enable patients to discuss DRPs in a timely manner with a health care provider, as it can provide faster contact between patients and health care providers without waiting or travel time. In addition, text messaging can be an addition to the already available communication channels, such as contact via telephone. Research demonstrated that offering multiple communication channels is important in providing patients with a suitable channel for discussing individual DRPs [12]. Finally, text messaging seems more efficient compared with phone calls, as shown by diabetes self-management support via text messaging requiring less staff time than via phone [13]. Furthermore, text messaging has been shown to be effective and feasible as an intervention to manage, for instance, blood pressure [14], adherence in type 2 diabetes [15], and postoperative pain [16]. However, these studies evaluated text messaging only as a channel for communication at the initiative of the health care provider to instruct patients (eg, by sending reminders regarding adherence) and provide health-related information to patients, and in most cases, only encompassed one-way texting from health care provider to patient (ie, patients could not respond to text messages).

As patients fulfill a key role in timely identifying potentially clinically relevant DRPs, this study opts for text messaging at the initiative of the patient, in which patients can contact their pharmacist when DRPs arise to facilitate timely resolution. This potentially provides a personalized and quick channel to raise, discuss, and resolve DRPs effectively. As patient-initiated text messaging with a pharmacist to address and resolve DRPs has not been evaluated, it is important to first study the feasibility of this functionality from the perspectives of both patients and pharmacists before its effect on reducing DRPs can be studied and large-scale implementation can be considered. Therefore, this study aims to assess the feasibility of text messaging between patients with inflammatory rheumatic diseases and pharmacists at the initiative of the patient when experiencing DRPs.

## Methods

### Study Design

A prospective feasibility study was conducted at the outpatient pharmacy of the Sint Maartenskliniek in the Netherlands between August 2023 and February 2024.

### Ethical Considerations

The Medical Research Ethics Committee of Arnhem-Nijmegen, the Netherlands, reviewed this study and waived official ethical approval (case 2023‐16580) as this study was deemed not subject to the Medical Research Involving Human Subjects Act. All participants gave informed consent digitally before commencement of the study (Multimedia Appendix 1). All participant data were anonymized to protect participant privacy and were stored on a secure environment only accessible to the researchers. Participants did not receive compensation for participation.

### Participants

Adult patients (≥18 y) with an inflammatory rheumatic disease receiving at least 1 DMARD from the outpatient pharmacy were eligible for inclusion. In addition, pharmacists covering pharmacy day shifts during the study period were eligible for participation. Pharmacists conducted study activities as part of their daily routine and gave consent verbally.

As the number and types of DRPs are likely dependent on the type of DMARDs patients use, as well as on the duration of their treatment [17], purposive sampling was performed using the following criteria: (1) biological disease-modifying antirheumatic drugs (bDMARDs), conventional synthetic DMARDS or targeted synthetic DMARDS; (2) patients initiating a new DMARD (new users) or patients using a specific DMARD for at least 6 months before inclusion (existing users). Although previous studies regarding sample sizes in feasibility studies advise sample sizes ranging from 12 to 35 participants per group [18,19], we aimed to include 60 participants (30 new and 30 existing users) distributed evenly among the 3 groups of DMARDs.

New users were selected from the pharmacy information system and were defined as patients who got their DMARD dispensed for the first time in the 2 weeks before inclusion. For bDMARDs, only bDMARD-naïve patients (ie, patients starting their first bDMARD ever) were eligible for participation, as switching between bDMARDs is common and DRPs reported by patients usually differ minimally between bDMARDs.

Existing users were selected from all patients who received a DMARD from the outpatient pharmacy in the last 3 months before the study started and those who had been using that specific DMARD for at least 6 months before the study started. A random sample was then taken from which patients were purposively approached for inclusion.

Inclusion was performed by telephone. During this phone call, patients received a verbal explanation of the study content. Patients willing to participate received written study information and a digital informed consent form. Participation started after informed consent was given.

### Intervention

The intervention used in this study was text messaging with a pharmacist at the initiative of the patient. This enables patients to report DRPs directly to a pharmacist. Participants were able to access the text messaging functionality by securely logging in to the hospital’s patient portal either via the hospital’s website or a smartphone app before each use. Participants were asked to discuss questions or problems regarding their antirheumatic drugs with a pharmacist using text messaging for a period of 8 weeks and received reminders at the start of the study and every 2 weeks thereafter, to ensure exposure to the intervention. There was no restriction on the number of conversations participants were allowed to initiate. The service level (ie, the aimed maximum time between patients reporting a DRP and pharmacists responding) was set at 4 hours from the moment the question was asked, within working hours and days (Monday to Friday between 9 AM and 5 PM).

### Feasibility Evaluation: Outcome Measures

#### Overview

Feasibility was assessed using the framework for designing feasibility studies by Bowen et al [20]. This widely applied model describes feasibility in 8 areas of focus. For this study, five of these eight domains were assessed: (1) demand, (2) limited-efficacy testing, (3) implementation, (4) acceptability, and (5) practicality. Furthermore, 3 domains (adaptation, integration, and expansion) were not included in this study as these focus on implementation and scale-up of the intervention, which was not yet relevant for the intervention under investigation. The domains of the framework by Bowen et al [20] were assessed using the outcomes of interest and outcome measures that are presented in Table 1.

**Table 1.** Outcome measures per domain of Bowen’s framework for designing feasibility studies.

Bowen’s domain, outcome of interest, and outcome measures	Subject^a^
Demand	
Actual use	
D^b^: Number of interactions	—^c^
D: Number of unique DRPs^d^	—
D: Type of DRPs	—
Expressed interest	
Q^e^: Subjective quality of text messaging (user-version Mobile Application Rating Scale - section E) [21]	Pt^f^ and Ph^g^
Additional questions	
Q: Factors impacting future use of text messaging	Pt
Q: Factors ensuring future use of text messaging	Pt
Limited-efficacy testing	
Effects on key variables	
D: Number of DRPs solved	—
D: Number of DRPs in need of follow-up	—
D: Number of DRPs warranting HCP^h^ involvement	—
Implementation	
Degree of execution	
D: Response within service level	—
Resources needed to implement	
D: HCP’s time investment per conversation	—
Acceptability	
Satisfaction and appropriateness	
Q: Acceptability of text messaging (theoretical framework acceptability) [22]	Pt and Ph
Additional questions	
Q: Benefits and disadvantages of text messaging	Pt and Ph
Q: Types of DRPs acceptable or not acceptable to discuss using text messaging	Pt and Ph
Q: Preferred service level	Pt and Ph
Q: Preferred form of address	Pt
Q: Preferred alternative if text messaging was not available	Pt
Q: Fit of text messaging within current care	Pt and Ph
Q: Added value of text messaging to current care	Pt
Practicality	
Ability to carry out intervention activities	—
Q: System usability (Dutch version of the System Usability Scale) [23]	Pt and Ph
Additional questions	
Q: Positive and negative properties regarding ease of use of text messaging	Pt and Ph

a Outcome measures part of the questionnaire were answered by patients, pharmacists, or both.

b Outcome measures with prefix ‘D’ were collected by means of usage data.

c Not applicable.

d DRP: drug-related problem.

e Outcome measures with prefix ‘Q’ were collected by means of questionnaire data.

f Pt: patients.

g Ph: pharmacists.

h HCP: health care provider.

#### Demand

To measure demand for the intervention, the actual use of the intervention in terms of conversations and DRPs was assessed. In addition, the extent to which the intervention was likely to be used was assessed by determining participants’ expressed interest in using the intervention, as well as factors impacting future use.

#### Limited-Efficacy Testing

To measure the efficacy of the intervention, the effect of the intervention on relevant key variables was measured. This was by measuring the extent to which discussed DRPs could be solved, the number of DRPs that needed follow-up from a health care provider other than a pharmacist, and the number of DRPs warranting health care provider involvement (ie, did an individual DRP need to be discussed with a pharmacist, or could this be solved by, for example, a frequently asked questions [FAQ] page).

#### Implementation

To measure implementation, the degree of execution was assessed by determining protocol fidelity, defined as the extent to which pharmacists responded to conversations within the service level. In addition, the resources needed to execute the intervention were assessed by calculating pharmacists’ time investment per conversation.

#### Acceptability

To measure the acceptability of the intervention, satisfaction and appropriateness of text messaging with a pharmacist were assessed as part of the questionnaire sent to participants. In addition, participants’ intention to (continue) using the intervention, satisfaction, preferred service level, preferred way patients wish to be addressed, preferred alternative if text messaging was not available, and added value of the text messaging to current care were assessed.

#### Practicality

To measure practicality, participants’ ability to carry out intervention activities (ie, using the intervention) was assessed. For patients, this meant using text messaging to initiate conversations with pharmacists, and for pharmacists, this meant answering conversations initiated by patients.

### Measurement Instruments

#### Study Sample

The patient characteristics that were retrieved from the electronic health record were age, sex, diagnosis, disease duration, number of drugs prescribed by a rheumatologist, and type of DMARDs at the start of the study.

#### Demand

Actual use was measured from usage data by counting the number of interactions initiated by the participants, the number of DRPs raised (as multiple DRPs can be raised in a single conversation), and the types of DRPs. To this end, individual DRPs were categorized according to the DRP classification as used by Haegens et al [4]. One researcher categorized DRPs (LH), which were reviewed by a second researcher (VH), after which discrepancies were discussed until consensus was reached. In case patients had not responded to the question of whether a DRP had been answered satisfactorily, one researcher (LH) determined if the DRP was resolved, after which a second researcher (VH) reviewed these cases, and discrepancies were discussed until consensus was reached. Expressed interest was assessed by incorporating the questions of the validated User version of the Mobile App Rating Scale [21] (uMARS) section E regarding subjective app quality into the questionnaire sent to the participants, consisting of 4 questions answered on 5-point Likert scales (Multimedia Appendix 2), which are reported individually. Other sections of the uMARS were not incorporated in this questionnaire, as they aim to assess factors other than demand for the intervention or other domains that are not of interest in this study. In addition, participants were asked about factors that positively or negatively impacted their future use of text messaging and how future use of text messaging can be ensured.

#### Limited-Efficacy Testing

DRPs were regarded as solved either when participants answered a confirmatory question at the end of every conversation positively or, in the case participants did not answer this question. In total, 2 researchers (LLH and VJBH) determined if DRPs were solved separately, after which discrepancies were discussed until consensus was reached. The number of DRPs that involved follow-up with a health care provider other than the pharmacist, or contained advice to do so, was counted from usage data. For each DRP, it was determined if raised DRPs warranted health care provider involvement by 2 researchers (LLH and VJBH), determining if individual DRPs needed to be discussed with a pharmacist or could be solved by, for example, an FAQ page independently, after which discrepancies were discussed until consensus was reached.

#### Implementation

Degree of execution was assessed by determining if pharmacists responded to individual conversations within the predefined service level of 4 hours, as well as pharmacists’ average response time. To measure this, only working hours (ie, time between 9 AM and 5 PM on Monday to Friday) were counted, and hours outside working hours were not. Pharmacists’ time investment per conversation (ie, including time for researching answers and contacting other health care providers) was registered by the pharmacist responding to conversations using a stopwatch.

#### Acceptability

Acceptability was measured using the questionnaire from the theoretical framework of acceptability [22], which consists of questions targeting 8 acceptability constructs answered on 5-point Likert scales (Multimedia Appendix 2) and are reported individually. In addition, open-ended questions regarding the intention to (continue) using the intervention, participants’ satisfaction, the preferred service level (ie, within what timeframe do patients expect to receive an answer from a pharmacist), patients’ preferred form of address, what alternatives patients would use instead of text messaging, and the added value of the text messaging to current care were constructed based on the framework by Bowen et al [20] and its description of the acceptability domain. All questions regarding acceptability were constructed by one researcher (LH) and discussed among coauthors (LH, BB, and VH) until consensus was reached.

#### Practicality

Participants’ ability to carry out intervention activities was assessed by using the validated Dutch translation of the System Usability Scale (SUS) [23], which is a 10-item questionnaire scored on a 5-point Likert scale from “strongly disagree” to “strongly agree” (Multimedia Appendix 2) that results in a score between 0 and 100. These questions were supplemented with open-ended questions regarding the main benefits and disadvantages of text messaging with a pharmacist, the main positive and negative properties regarding the ease of use of text messaging, and the fit of text messaging with current care.

### Data Collection

Data on the feasibility of the intervention were collected either via a web-based questionnaire sent to all participants (both patients and pharmacists) or from usage data from the text messaging app. Furthermore, 2 separate questionnaires were constructed for participating patients and pharmacists. These questionnaires consisted of the same domains and predominantly differed in wording (eg, asking questions vs answering questions). Some questions, however, were not included in the questionnaire for pharmacists due to nonrelevance (Multimedia Appendix 2). Participants received a notification from an electronic data capture system via email with the invitation to complete the questionnaire and were reminded after 1 week if needed. Usage data were collected from the system that the text messaging is part of, by extracting relevant data regarding individual conversations into a separate database.

### Data Analysis

Participating patients were regarded as “active participants” if at least 1 conversation was initiated via text messaging and were otherwise regarded as “nonactive participants” and thus excluded from the analyses. Usage data and data from questionnaires were descriptively analyzed using STATA 17 (StataCorp LLC) [24]. Proportions were presented as percentages. Parametric data were presented as means with SDs; nonparametric data were presented as medians with IQRs. Questionnaires with standardized analysis were scored and reported accordingly.

## Results

### Population Characteristics

Of the 108 patients that approached for participation, 56% (n=60) of patients were included and signed informed consent with a median age of 58 (IQR 53-65) years, and 70% (n=42) were female (Table 2). The most frequent reasons reported by patients for not participating were not interested (n=13) or too busy (n=8). Of the 60 included patients, 75% (n=45) of patients (median age 57, IQR 52-65 y; and n=31, 69% female) raised at least 1 DRP via text messaging (hereinafter referred to as active participants) and received the questionnaire. Reasons for not actively participating were that participants forgot to ask questions or were unable to ask questions due to personal reasons. Of the active participants, 87% (n=39) completed the questionnaire fully (hereinafter referred to as responders), 4% (n=2) of active participants completed the questionnaire partly, of whom only the fully completed domains are reported. Furthermore, 5 pharmacists (median age 41, IQR 26-47 y; and n=1, 20% female) participated in this study by responding to the text messages.

**Table 2.** Population characteristics.

Characteristic	Active participants^a^ (n=45)	Nonactive participants^b^ (n=15)
Sex, n (%)		
Female	31 (70)	10 (64)
Age (y), median (IQR)	57 (52‐65)	61 (54‐65)
Diagnosis, n (%)		
Rheumatoid arthritis	24 (53)	11 (73)
Psoriatic arthritis	10 (22)	1 (7)
Axial spondyloarthritis	6 (13)	1 (7)
Other	5 (11)	1 (7)
Disease duration (y), median (IQR)	6 (2-10)	9 (2-15)
Drugs prescribed by rheumatologist, median (IQR)	3 (4-5)	3 (5-6)
DMARD^c^ group of inclusion, n (%)		
csDMARD^d^	16 (36)	7 (47)
tsDMARD^e^	11 (24)	5 (33)
bDMARD^f^	18 (40)	2 (13)
Experience category, n (%)		
New user	23 (51)	7 (47)
Experienced user	22 (49)	8 (53)

a Active participants are defined as included patients that discussed at least one drug-related problem during the study using text messaging.

b Nonactive participants are defined as included patients that did not raise a drug-related problem during the study.

c DMARD: disease-modifying antirheumatic drug.

d csDMARD: conventional synthetic disease-modifying antirheumatic drug.

e tsDMARD: targeted synthetic disease-modifying antirheumatic drug.

f bDMARD: biological disease-modifying antirheumatic drug.

### Demand

During the study, 45 participants initiated 133 conversations via text messaging containing 158 unique DRPs (ie, DRPs that had not been raised before by individual participants), with a median of 3 (IQR 2-4) unique DRPs per participant. DRPs reported concerned information needs (n=42, 27%), (suspected) side effects (n=36, 23%), problems regarding medication management (n=34, 22%), logistics (n=14, 9%), medication concerns (n=13, 8%), medication effectiveness (n=9, 6%), contraindications (n=5, 3%), and others (n=5, 3%).

In total, 41 patients (91%) and 5 pharmacists (100%) completed the questions regarding demand. Demand was measured as the expressed interest or intention to use text messaging (uMARS section E). Both patients and pharmacists scored text messaging overall with a median of 4 (IQR 4-5) out of 5 stars. Out of 41 patients, 37 (90%) patients would recommend text messaging to others who could benefit from this service, and all 5 pharmacists would recommend text messaging to others. Furthermore, 63% (26/41) of patients would use text messaging at least once a year, and 32% (13/41) would consider paying for text messaging.

To ensure future use, 39% (16/41) of patients mentioned promotion of text messaging as a means to discuss DRPs, 20% (8/41) providing good instructions regarding and support with text messaging, and 17% (7/41) reducing the number of steps to navigate to the text messaging within the online patient portal as improvements. Furthermore, 34% (14/41) of patients mentioned quick answers, 27% (11/41) mentioned text messaging being easy to use, and 15% (6/41) receiving clear answers to questions as factors that would positively impact future use. Factors negatively impacting future use included text messaging being impractical (2/41, 5%) and impersonal (2/41, 5%).

### Limited-Efficacy Testing

Of the 158 unique DRPs raised by patients, 58% (n=92) were solved according to patient confirmation, and the remaining 42% were solved according to pharmacist determination. In total, 5 reported DRPs were followed up in consultation with other health care providers: 3 with a rheumatologist, 1 with a specialized rheumatology nurse, and 1 with a pharmacy technician. In addition, 17 DRPs resulted in pharmacists advising follow-ups, mainly with the patient’s rheumatologist (n=12). It was determined that 77% (122/158) of DRPs required a health care provider, and 23% (36/158) could have been solved without health care provider involvement. Within DRPs that required health care provider involvement, the most common types of DRPs were suspected side effects (32/122, 26%), medication management (30/122, 25%), and information needs (16/122, 13%). DRPs that did not require health care provider involvement consisted of information needs (26/36, 72%), suspected side effects (4/36, 11%), and medication management (4/36, 11%). All types of DRPs were determined to require health care provider involvement for the majority of DRPs within their type (ranging from 30/34, 88% to N=13, 100%), except for DRPs regarding information needs, for which 2 out of 3 DRPs did not require health care provider involvement.

### Implementation

Of the 133 conversations initiated by patients, 116 (87%) conversations were answered within the predefined service level of 4 hours. The median time until response was 80 (IQR 34-153) minutes with a total range of 0 minutes to 39.2 hours within opening hours of the pharmacy. The pharmacists’ median time investment per conversation was 4:21 (IQR 2:15-7:41) minutes for DRPs that required health care provider involvement and 3:32 (IQR 2:39-6:41) minutes for DRPs that did not require health care provider involvement, which did not differ significantly (*P*=.10).

### Acceptability

**Figure 1.**
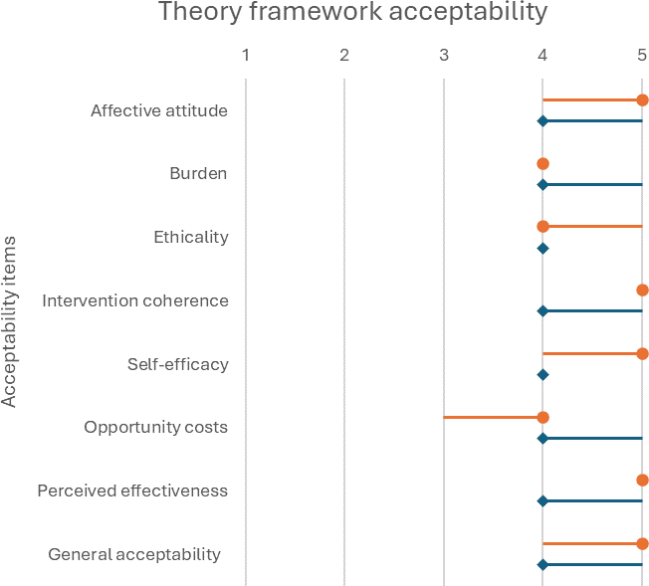
Patients’ (orange) and pharmacists’ (blue) acceptability scores for text messaging between patients and pharmacists to discuss drug-related problems at the initiative of the patient, measured using the theoretical framework acceptability [22]. Dots present median values, lines indicate IQRs.

From the 45 active participants, 39 patients (87%) and all 5 pharmacists completed the questions regarding acceptability. Patients scored acceptability of the intervention with a median score of 4 (IQR 4-5) out of 5, and pharmacists with a median of 5 (IQR 4-5) (Figure 1).

The top 3 most-mentioned advantages and disadvantages regarding the acceptability of text messaging mentioned by participating patients and pharmacists, as well as types of DRPs that are acceptable and not acceptable to discuss via text messaging, are presented in Table 3.

**Table 3.** Top 3 (where available) most-mentioned responses regarding advantages and disadvantages of text messaging, as well as types of DRPs acceptable and not acceptable for discussing via text messaging.

Questionnaire item, respondent, and response	Value, n (%)
Advantages regarding acceptability of text messaging	
Patients (n=39)	
Quick answers	21 (54)
Clear answers	9 (17)
No waiting times	7 (13)
Pharmacists (n=5)	
Accessibility of contact between patient and pharmacist	4 (80)
Sufficient time to formulate answer	3 (60)
Disadvantages regarding acceptability of text messaging	
Patients (n=39)	
Difficulties with logging in	4 (10)
Too many steps to reach text messaging	2 (5)
Less personal than contact via telephone	1 (3)
Pharmacists (n=5)	
Answering messages not yet embedded in daily routine	2 (40)
Contact becoming too accessible might lead to unnecessary contact	1 (20)
Questions need to be carefully answered, as these are in writing	1 (20)
Types of DRPs acceptable for discussing via text messaging	
Patients (n=39)	
Side effects	9 (23)
Everything regarding medication	4 (10)
Medication intake	3 (8)
Pharmacists (n=5)	
Factual questions regarding information	3 (60)
(Mild) side effects	2 (40)
Practical problems such as medication intake	2 (40)
Types of DRPs not acceptable for discussing via text messaging	
Patients (n=39)	
Questions for treating physician	1 (3)
Questions in need of in-person answer	1 (3)
Pharmacists (n=5)	
Insufficient medication effect	1 (20)
Allergies	1 (20)
Acute side effects	1 (20)
Emotionally-loaded questions	1 (20)

In total, 37 out of 45 patients (82%) and all pharmacists (N=5, 100%) completed the additional service-related questions. Regarding the preferred service level (ie, within what timeframe do patients prefer to receive a response to their text message), the majority of patients preferred a response within 4 (17/37, 46%) or 24 hours (12/37, 32%), with 2 patients (5%) indicating that the service level is dependent on individual DRPs. Pharmacists preferred a service level of 24 hours (2/5, 40%), 8 hours (1/5, 20%), or 4 hours (1/5, 20%), with 2 pharmacists (40%) suggesting letting the patient indicate the service level per DRP. Patients preferred to be addressed informally (15/37, 41%), formally (6/37, 16%), or did not have a preference (14/37, 38%). The majority of patients (24/37, 65%) would have called the pharmacy if text messaging were not available, and 11% (4/37) indicated having contacted their health care provider but did not specify in which way. Another 11% (4/37) of patients indicated they would wait until the next consultation to discuss their DRP, and another 5% (2/37) would not discuss their DRP at all. Patients scored the extent to which text messaging fits into current care with a median of 4 (IQR 4-5), whereas pharmacists scored fit with a median of 4 (IQR 3-4). Pharmacists mentioned the inability to redirect questions to other health care providers and the fact that text messaging is not well-integrated into the existing information technology systems currently in use as disadvantages. In general, the added value of text messaging according to patients included quick answer to questions (7/37, 19%), having an additional way to ask questions (3/37, 8%), easy accessibility of contacting a pharmacist (2/37, 5%), and having an additional option for support when starting with new medication (2/37, 5%).

### Practicality

Out of 45 patients, 39 (87%) and all 5 pharmacists (100%) completed the questions regarding practicality. The mean SUS score was 72 (SD 18) among patients, with 22 participants (57%) rating usability as good, excellent, or best imaginable. Mean SUS score was 81 (SD 16) among pharmacists, with 4 pharmacists (80%) rating usability as good, excellent, or best imaginable. The most-mentioned responses to the additional open-ended questions regarding practicality can be found in Table 4.

**Table 4.** Most-mentioned responses to additional open-ended questions regarding practicality.

Questionnaire item, respondent, and response	Value, n (%)
Properties positively influencing ease of use of text messaging	
Patients (n=39)	
Quick answer	14 (36)
Time- and place-independent	7 (18)
Easy to use	5 (13)
No waiting time	5 (13)
Pharmacists (n=5)	
Easy to use	3 (60)
Privacy-friendly	1 (20)
Time to formulate answers	1 (20)
Properties negatively influencing ease of use of text messaging	
Patients (n=39)	
Too many steps to reach text messaging within online patient-portal	8 (21)
Difficulties with logging in	6 (15)
Image size too small on mobile	3 (8)
Pharmacists (n=5)	
Absence of notifications of new messages	2 (40)
Less personal contact	1 (20)
Uncertainty as to whether DRP has been solved	1 (20)

## Discussion

### Principal Findings

In this study, the feasibility of text messaging at the initiative of the patient to discuss DRPs directly with pharmacists was examined. Text messaging between patient and pharmacist resulted in 158 unique DRPs in 133 conversations over an 8-week period. Pharmacists responded to all messages with advice to solve the problem or an answer to the question. All DRPs were solved, and most (116/133, 87%) were within the promised service level, and 14% (22/158) of DRPs resulted in follow-up or advised follow-up by health care providers other than pharmacists.

Both patients and pharmacists mentioned good accessibility of information and contact between patient and pharmacist as important factors for the acceptability of text messaging, which is also confirmed by other studies where accessibility was regarded as an important factor in communication between patient and pharmacist [25,26] as well as a driver for patients, preferring more accessible channels [12,27]. However, 1 pharmacist in our study noted that too easily accessible contact between patient and health care provider could lead to unnecessary contact (ie, contact regarding DRPs that do not warrant health care provider involvement) and thus an increased workload, which is a concern found in several other studies regarding the growing application of eHealth and its relation to health care providers’ workload [28,29]. However, data on the actual magnitude of this effect is lacking [30] and should thus be further researched.

In a study by Turcotte et al [25], patients mentioned the feeling of pharmacists being able to devote more time to answering their questions, which is in line with the pharmacists in our study mentioning having sufficient time to answer individual questions from patients when compared with current care. In a study by Zafar et al [26], patients were unsure about what type of questions were appropriate to ask via text messaging. Although patients in our study named several types of DRPs they find suitable to discuss, some differences exist between what patients and pharmacists view as appropriate DRPs to discuss via text messaging, with pharmacists making a distinction between the severity of side effects that patients do not make, which is important to align in managing patients’ and pharmacists’ expectations.

Although comparable studies did not employ the SUS, usability was rated “good” or “best imaginable” by the majority of patients and pharmacists, and both scored above the average SUS value of 68 [31]. Regarding the ease of using text messaging, which was in general mentioned as a positive factor driving acceptability, patients mentioned time- and place-independence and shorter waiting times of text messaging as positive properties. The SUS scores possibly find their origin in the comparability between the text messaging applied in this study with online and app-based consumer text messaging services. As some downsides regarding the practicality of the text messaging app used in this study were mentioned, it might be beneficial to organize and offer text messaging in a comparable manner to consumer apps to increase ease of use and thus acceptability, for example, by easing or removing the login procedure.

The top 3 most common types of patient-reported DRPs were information needs (42/158, 27%), (suspected) side effects (36/158, 23%), and medication management (34/158, 22%). These numbers are comparable with previous research regarding types of DRPs discussed with patients with inflammatory rheumatic diseases via 4 biweekly phone calls, in which (suspected) side effects accounted for 28% (85/308) and medication management for 26% (80/308) [4]. In our study, 27% (42/158) of DRPs were related to information needs, which is considerably higher than the 4% (13/308) of DRPs in the aforementioned study, which could be explained by the fact that DRPs in this study were patient-initiated, while the referred study assessed DRPs in health care provider–initiated conversations. In addition, the conceivability that patients regard information-related DRPs as more suitable to discuss via text messaging than via telephone might also have contributed to this. Although one might conclude that DRPs related to information needs could be solved by channels without health care provider involvement as this mainly pertains to information provision, 77% (122/158) of all DRPs were determined to warrant health care provider involvement, including 38% (16/42) of all DRPs related to information needs which mostly concerned needs for information regarding individual patient’s treatment (eg, questions regarding safety of alternative medicine in combination with current therapy).

In practice, questions and DRPs can be answered and solved through various communication channels, such as during consultations, telephone calls, or with an FAQ page, for example. In this context, text messaging is a valuable addition to currently available channels, offering patients with a wide array of channels to discuss and resolve DRPs with high quality and efficiency, corresponding with patients’ individual needs [12].

### Implications

Patients in this study were generally positive regarding the feasibility of text messaging with pharmacists to discuss DRPs. The fact that 65% (24/37) of patients indicated that they would have raised their DRPs via telephone if text messaging had not been available implies that patients view text messaging as a suitable channel for raising DRPs in which health care provider involvement is warranted from the patient’s perspective. In addition, 16% (6/37) of patients indicated that they would wait for the next consultation to raise a DRP if text messaging were not available, indicating DRPs possibly remain unraised and thus unsolved for a longer period of time. Although presumably part of these DRPs can wait until the next consultation, another part can lead to negative health consequences if not solved in a timely manner. Offering text messaging in addition to the currently available channels could lead to more DRPs raised, as well as in an earlier stage, which might therefore prevent negative health consequences associated with DRPs, as well as save time during consultations normally dedicated to such problems. Although all DRPs raised by patients were solved, either by patient confirmation or expert determination, the effect of this on patients’ health outcomes was not assessed. Therefore, future research should aim to quantitatively assess the impact of the intervention on patients’ clinical outcomes, such as medication adherence or improvement of health, as pharmacist interventions aimed at lowering DRPs seem to be able to improve clinical outcomes in other settings [32].

For health care providers, implementing text messaging can have several implications. First, the actual demand of text messaging in a large population will have an impact on its feasibility, as pharmacists must be able to incorporate the workload associated with text messaging into their daily routine. To this end, real-life demand needs to be further researched. As demand for text messaging is also related to the demand for other available channels, this should be researched in the context of all available channels rather than for text messaging alone. Second, although patients mentioned some advantages of text messaging over contacting a pharmacist via telephone, the extent to which text messaging can reduce the number of contacts via telephone or other channels and save time and resources is still unknown and should be researched in a trial with sufficient follow-up and a subsequent cost-benefit analysis. Third, although text messaging might initially increase pharmacists’ workload, the potential of text messaging to save time and resources further down the chain (eg, by saving time during consultations) should be researched to determine the net decrease (or increase) in workload. Fourth, to avoid offering text messaging from leading to an additional workload, it is important to prevent (intentional or unintentional) inappropriate use of text messaging (ie, raising DRPs that could be solved without health care provider involvement, for example, using an FAQ), which could be applicable for approximately 1 in 4 DRPs raised in this study that did not warrant health care provider involvement. Therefore, it is important to communicate to patients what DRPs are suitable to discuss via text messaging and what DRPs better suit different channels, or direct patients to the most suitable channel based on the subject of their DRP, for which DRPs warranting health care provider involvement in this study could be a first indication.

In terms of technological feasibility, several implications need to be considered to improve implementability and future uptake. Patients indicated that ease of use should be optimal for them to use text messaging most optimally. To this end, minor improvements should be made (eg, to the logging in procedure and the number of steps patients need to take) to be able to raise a DRP within the online patient portal. However, overall feasibility was scored as high, and 90% (37/41) of participants would recommend text messaging to others. Importantly, although pharmacists scored system usability above average and higher than patients, the fit with current care was scored lower than patients. Therefore, it is essential to look further into seamless integration of text messaging into the existing pharmacy systems and workflow, as well as ensuring seamless communication with other health care providers, to maximize its potential and ensure future use.

### Limitations

Some limitations should be acknowledged. First, patients were reminded to ask a question at least once every 2 weeks to ensure user experience before completing the questionnaire. This could have led to DRPs being made up for study purposes, and thus we cannot conclude on the real-life demand that exists for text messaging from the patients’ side in the researched population and setting. Second, 2 out of 5 pharmacists who answered patient messages were also involved in the conception of this study and as coauthors of this paper. This could have led to bias regarding outcome measures that included pharmacists’ involvement, such as answering DRPs within the promised service level. However, as feasibility was researched in the context of current practice, the involvement of participating pharmacists in the conception of outcome measures was an added value, as they were able to include metrics that are needed for assessing feasibility and deciding future research and implementation. Third, selection bias might have been present in the study sample; for example, more digitally inclined or eHealth-literate patients could have agreed to participate than digitally-averse or eHealth-illiterate patients. This possibly led to an overestimation of the feasibility of text messaging from the patient’s perspective, as these patients might have a more positive attitude toward digital tools and eHealth in general. Although 1 in 4 patients did not actively participate, these patients did not significantly differ from active participants in terms of sex, age, diagnosis, disease years, or prescribed DMARD group. Fourth, the response rate to the question of whether a DRP was answered sufficiently was low, with a nonresponse rate of 42%, resulting in a large number of DRPs determined to be answered sufficiently by the pharmacist’s determination. Despite meticulous determination by 2 researchers (LLH and VJBH), this determination might not be as precise as patient confirmation. The relatively low response rate might be caused by the fact that the need to indicate if a question was answered satisfactorily after the conversation is concluded might not be intuitive and is normally not present in face-to-face conversations. Finally, the generalizability of the study is limited. As this study was performed in a specialized center rather than a general hospital and on a limited number of chronic conditions, standards of current (digital) care might differ from those in general hospitals and other chronic conditions. This can result in a different point of reference and thus make this study’s findings not fully generalizable to other settings.

### Conclusion

To conclude, 2-way text messaging between patients and pharmacists for discussing DRPs seems feasible from the patients’ and pharmacists’ perspectives. Furthermore, participants used text messaging primarily for raising DRPs, warranting health care provider involvement, which shows the potential of text messaging as an efficient addition to currently available information and communication channels if correctly integrated and intuitively usable. However, the impact of actual demand in a larger population on the feasibility for health care providers, as well as the impact on prevention of possible negative health consequences of DRPs, should be researched before full-scale implementation.

## Supplementary material

10.2196/66514 Multimedia Appendix 1 Blank informed consent form in Dutch.

10.2196/66514 Multimedia Appendix 2 Questionnaires.

## Abbreviations

bDMARD: biological disease-modifying antirheumatic drug
DMARD: disease-modifying antirheumatic drug
DRP: drug-related problem
FAQ: frequently asked question
SUS: System Usability Scale
uMARS: user-version of the Mobile Application Rating Scale

## References

[1] Abbasi M, Mousavi MJ, Jamalzehi S (2019). Strategies toward rheumatoid arthritis therapy; the old and the new. J Cell Physiol.

[2] Vrijens B, De Geest S, Hughes DA (2012). A new taxonomy for describing and defining adherence to medications. Br J Clin Pharmacol.

[3] Gil-Guillen VF, Balsa A, Bernárdez B (2022). Medication non-adherence in rheumatology, oncology and cardiology: a review of the literature of risk factors and potential interventions. Int J Environ Res Public Health.

[4] Haegens LL, Huiskes VJB, Smale EM, Bekker CL, van den Bemt BJF (2023). Drug-related problems reported by patients with rheumatic diseases: an observational study. BMC Rheumatol.

[5] Leendertse AJ, Van Den Bemt PMLA, Poolman JB, Stoker LJ, Egberts ACG, Postma MJ (2011). Preventable hospital admissions related to medication (HARM): Cost analysis of the HARM study. Value Health.

[6] Magdelijns FJH, Stassen PM, Stehouwer CDA, Pijpers E (2014). Direct health care costs of hospital admissions due to adverse events in the Netherlands. Eur J Public Health.

[7] Huiskes VJB, Cramer-van der Welle CM, van den Ende CHM (2020). Communication about drug-related problems (DRPs) during patients’ visits to Dutch physicians and pharmacies. Health Commun.

[8] Mathijssen EG, Vriezekolk JE, Eijsbouts AM, van den Hoogen FH, van den Bemt BJ (2018). Support needs for medication use and the suitability of eHealth technologies to address these needs: a focus group study of older patients with rheumatoid arthritis. Patient Prefer Adherence.

[9] Benefits of eHealth. Government of the Netherlands.

[10] Kwint HF, Faber A, Gussekloo J, Bouvy ML (2012). The contribution of patient interviews to the identification of drug-related problems in home medication review. J Clin Pharm Ther.

[11] Kari H, Kortejärvi H, Airaksinen M, Laaksonen R (2018). Patient involvement is essential in identifying drug‐related problems. Brit J Clinical Pharma.

[12] Haegens LL, Huiskes VJB, van der Ven J, van den Bemt BJF, Bekker CL (2023). Factors influencing preferences of patients with rheumatic diseases regarding telehealth channels for support with medication use: qualitative study. JMIR Form Res.

[13] Greenwood DA, Hankins AI, Parise CA, Spier V, Olveda J, Buss KA (2014). A comparison of in-person, telephone, and secure messaging for type 2 diabetes self-management support. Diabetes Educ.

[14] Zhai P, Hayat K, Ji W (2020). Efficacy of text messaging and personal consultation by pharmacy students among adults with hypertension: randomized controlled trial. J Med Internet Res.

[15] Gautier JF, Boitard C, Michiels Y, Raymond G, Vergez G, Guedon G (2021). Impact of personalized text messages from pharmacists on medication adherence in type 2 diabetes in France: A real-world, randomized, comparative study. Patient Educ Couns.

[16] Buck C, Keweloh C, Bouras A, Simoes EJ (2021). Efficacy of short message service text messaging interventions for postoperative pain management: systematic review. JMIR Mhealth Uhealth.

[17] Grove ML, Hassell AB, Hay EM, Shadforth MF (2001). Adverse reactions to disease-modifying anti-rheumatic drugs in clinical practice. QJM.

[18] Julious SA (2005). Sample size of 12 per group rule of thumb for a pilot study. Pharm Stat.

[19] Teare MD, Dimairo M, Shephard N, Hayman A, Whitehead A, Walters SJ (2014). Sample size requirements to estimate key design parameters from external pilot randomised controlled trials: a simulation study. Trials.

[20] Bowen DJ, Kreuter M, Spring B (2009). How we design feasibility studies. Am J Prev Med.

[21] Stoyanov SR, Hides L, Kavanagh DJ, Wilson H (2016). Development and validation of the user version of the Mobile Application Rating Scale (uMARS). JMIR Mhealth Uhealth.

[22] Sekhon M, Cartwright M, Francis JJ (2022). Development of a theory-informed questionnaire to assess the acceptability of healthcare interventions. BMC Health Serv Res.

[23] Ensink CJ, Keijsers NLW, Groen BE (2024). Translation and validation of the System Usability Scale to a Dutch version: D-SUS. Disabil Rehabil.

[24] (2021). Stata statistical software: release 17.

[25] Turcotte V, Chagnon A, Guénette L (2021). Experience and perspectives of users and non-users of the Ask your pharmacist teleconsultation platform. Explor Res Clin Soc Pharm.

[26] Zafar SN, Hazlewood G, Dhiman K (2024). “How are you?” Perspectives from patients and health care providers of text messaging to support rheumatoid arthritis care: a thematic analysis. ACR Open Rheumatol.

[27] van der Ven J, van den Bemt BJF, van Dijk L (2023). Preferences of patients with musculoskeletal disorders regarding the timing and channel of eHealth and factors influencing its use: mixed methods study. JMIR Hum Factors.

[28] MacLure K, Stewart D, Strath A (2014). A systematic review of medical and non-medical practitioners’ views of the impact of eHealth on shared care. Eur J Hosp Pharm.

[29] Ariens LF, Schussler-Raymakers FM, Frima C (2017). Barriers and facilitators to eHealth use in daily practice: perspectives of patients and professionals in dermatology. J Med Internet Res.

[30] Stevens WJM, van der Sande R, Beijer LJ, Gerritsen MG, Assendelft WJ (2019). eHealth apps replacing or complementing health care contacts: scoping review on adverse effects. J Med Internet Res.

[31] Bangor A, Kortum P, Miller J (2009). Determining what individual SUS scores mean: adding an adjective rating scale. J Usability Stud.

[32] Ayhan YE, Aksoy M, Abdulrahman Y (2025). Evaluation of the effect of management of drug-related problems on clinical outcomes of pulmonary embolism outpatients: a randomized controlled trial. J Clin Med.

