# Supplementary material for: Text Messaging Between Patients With Inflammatory Rheumatic Diseases and Pharmacists to Solve Drug-Related Problems: Prospective Feasibility Study
Source: JMIR Hum Factors. 2025 Oct 8;12:e66514. doi: 10.2196/66514 (PMC12507129; doi:10.2196/66514)
Supplement: Multimedia Appendix 1 [file humanfactors-v12-e66514-s001.docx]

**Toestemmingsformulier voor deelnemers**

Behorende bij:

**Onderzoek naar het chatten met een apotheker over vragen of problemen met uw medicijn**

- Ik heb de informatiebrief en de brochure ‘Medisch-wetenschappelijk onderzoek: algemene informatie voor de proefpersoon’ gelezen. Ook kon ik vragen stellen. Mijn vragen zijn goed genoeg beantwoord. Ik had genoeg tijd om te beslissen of ik meedoe.
- Ik weet dat meedoen vrijwillig is. Ook weet ik dat ik op ieder moment kan beslissen om toch niet mee te doen met het onderzoek. Of om ermee te stoppen. Ik hoef dan niet te zeggen waarom ik wil stoppen.
- Ik geef de onderzoekers toestemming om mijn gegevens te verzamelen en gebruiken. De onderzoekers doen dit alleen om de onderzoeksvraag van dit onderzoek te beantwoorden. De onderzoekers zullen mijn gegevens strikt vertrouwelijk en anoniem behandelen.
- Ik weet dat voor de controle van het onderzoek sommige mensen al mijn gegevens kunnen inzien. Die mensen staan in deze informatiebrief. Ik geef deze mensen toestemming om mijn gegevens in te zien voor deze controle.

o Ja, ik wil meedoen aan het onderzoek.

o Nee, ik wil niet meedoen aan dit onderzoek.
