# Supplementary material for: Text Messaging Between Patients With Inflammatory Rheumatic Diseases and Pharmacists to Solve Drug-Related Problems: Prospective Feasibility Study
Source: JMIR Hum Factors. 2025 Oct 8;12:e66514. doi: 10.2196/66514 (PMC12507129; doi:10.2196/66514)
Supplement: Multimedia Appendix 2 [file humanfactors-v12-e66514-s002.docx]

**Appendix A.** English translation questionnaires

*Questionnaire for patients*

**Part 1**

Theoretical Framework Acceptability

Questions were answered on 5-point Likert-scales.

1. How comfortable did you feel while text messaging with a pharmacist about questions or problems with your medication for your rheumatic disease?
2. How much effort did it take to text message with a pharmacist about questions or problems with your medication for your rheumatic disease?
3. How fair do you think text messaging with a pharmacist about questions or problems with your medication for your rheumatic disease is?
4. Text messaging with a pharmacist helps me to resolve questions or problems with my medication for my rheumatic disease.
5. I understand how text messaging with a pharmacist can help me resolve questions or problems with my medication for my rheumatic disease.
6. How confident do you feel while text messaging with a pharmacist about questions or problems with your medication for your rheumatic disease?
7. Text messaging with a pharmacist about questions or problems with my medication for my rheumatic disease interfered with my other priorities.
8. How acceptable did you feel it was to resolve questions or problems with your medication for your rheumatic disease by text messaging with a pharmacist?

Additional open-ended questions

1. What are the main reasons you are satisfied with text messaging with a pharmacist about questions or problems with your medication for your rheumatic disease?
2. What are the two main reasons you are dissatisfied with text messaging with a pharmacist about questions or problems with your medication for your rheumatic disease?

User-version of the Mobile Application Rating Scale – section E

Questions were answered on 5-point Likert-scales.

1. Would you recommend text messaging with a pharmacist about questions or problems with medication for a rheumatic disease to people who could benefit from it??
2. How often do you think you would use text messaging with a pharmacist about questions or problems with your medication for your rheumatic disease in the next 12 months, if this were applicable to you?
3. Are you willing to pay for cha text messaging with a pharmacist?
4. How would you rate text messaging with a pharmacist out of five stars?

Additional open-ended questions

1. Are there drug-related problems that you would like to discuss with a pharmacist via text messaging?
2. Are there drug-related problems that you would rather not discuss with a pharmacist via text messaging?
3. In the future, would you discuss the same kind of problems and questions you have discussed via text messaging with a pharmacist during the study via text messaging again?
   1. If yes, why?
   2. If no, why not? And what alternative channel would you use?

**Part 2**

Additional open-ended questions

1. What are the two main advantages of text messaging with a pharmacist?
2. What are the two main disadvantages of text messaging with a pharmacist?

System Usability Scale

Questions were answered on 5-point Likert scales.

1. I think I would like to use this system frequently.
2. I found the system unnecessarily complex.
3. I thought the system was easy to use.
4. I think that I would need the support of a technical person to be able to use this system.
5. I found the various functions in the system were well integrated.
6. I thought there was too much inconsistency in this system.
7. I imagine that most people would learn to use this system very quickly.
8. I found the system very awkward to use.
9. I felt very confident using the system.
10. I needed to learn a lot of things before I could get going with this product.

Additional open-ended questions

1. What are the two most important positive features regarding the ease of use of text messaging with a pharmacist?
2. What are the two most important negative features regarding the ease of use of text messaging with a pharmacist?
3. Did you encounter any practical or technical problems while using text messaging with a pharmacist? If so, which ones?
4. Do you have any other comments about text messaging with a pharmacist to discuss drug-related problems?

*Questionnaire for pharmacists*

**Part 1**

Theoretical Framework Acceptability

Questions were answered on 5-point Likert-scales.

1. How comfortable did you feel while text messaging with patients about questions or problems with their medication for a rheumatic disease?
2. How much effort did it take to text message with patients about questions or problems with their medication for a rheumatic disease?
3. How fair do you think text messaging with patients about questions or problems with their medication for a rheumatic disease is?
4. Text messaging with patients helps me to resolve questions or problems with their medication for a rheumatic disease.
5. I understand how text messaging with patients can help me resolve questions or problems with their medication for a rheumatic disease.
6. How confident do you feel while text messaging with patients about questions or problems with their medication for a rheumatic disease?
7. Text messaging with patients about questions or problems with their medication for a rheumatic disease interfered with my other priorities.
8. How acceptable did you feel it was to resolve questions or problems with patients’ medication for a rheumatic disease via text messaging?

Additional open-ended questions

1. What are the main reasons you are satisfied with text messaging with patients about questions or problems with their medication for a rheumatic disease?
2. What are the main reasons you are dissatisfied with text messaging with patients about questions or problems with their medication for a rheumatic disease?

User-version of the Mobile Application Rating Scale – section E

Questions were answered on 5-point Likert-scales.

1. Would you recommend text messaging with patients about questions or problems with medication for a rheumatic disease to people who could benefit from it??

Additional open-ended questions

1. Are there drug-related problems that you would like to discuss with patients via text messaging?
2. Are there drug-related problems that you would rather not discuss with patients via text messaging?

**Part 2**

Additional open-ended questions

1. What are the two main advantages of text messaging with a pharmacist?
2. What are the two main disadvantages of text messaging with a pharmacist?

System Usability Scale

Questions were answered on 5-point Likert scales.

1. I think I would like to use this system frequently.
2. I found the system unnecessarily complex.
3. I thought the system was easy to use.
4. I think that I would need the support of a technical person to be able to use this system.
5. I found the various functions in the system were well integrated.
6. I thought there was too much inconsistency in this system.
7. I imagine that most people would learn to use this system very quickly.
8. I found the system very awkward to use.
9. I felt very confident using the system.
10. I needed to learn a lot of things before I could get going with this product.

Additional open-ended questions

1. What are the two most important positive features regarding the ease of use of text messaging with a pharmacist?
2. What are the two most important negative features regarding the ease of use of text messaging with a pharmacist?
3. Did you encounter any practical or technical problems while using text messaging with a pharmacist? If so, which ones?
4. Do you have any other comments about text messaging with patients to discuss drug-related problems?
